# Cervical Insufficiency Beyond Terminology: From Fixed Labels to Pregnancy-Specific Vulnerability in Personalized Maternal–Fetal Care

**DOI:** 10.3390/jpm16030149

**Published:** 2026-03-04

**Authors:** Moon-Il Park, Yong-Jin Park

**Affiliations:** 1 Department of Obstetrics and Gynecology, Cervical Insufficiency Center, Dongtan Jeil Women’s Hospital, Hwaseong 18450, Republic of Korea; 2 Department of Obstetrics and Gynecology, Hanyang University Hospital, Seoul 04763, Republic of Korea

**Keywords:** cervical insufficiency, preterm birth, personalized medicine, cervical vulnerability, risk stratification, pregnancy-specific trajectories, maternal–fetal medicine

## Abstract

Over the past two decades, the term cervical incompetence has largely been replaced by cervical insufficiency in clinical guidelines, reflecting efforts to avoid pejorative language and to acknowledge functional variability. However, despite this terminological evolution, the underlying conceptual framework has remained largely static, continuing to treat cervical insufficiency as a fixed anatomic defect inferred from obstetric history or single-point measurements. This Perspective argues that such a model inadequately explains the substantial clinical heterogeneity observed across and within pregnancies, limiting its usefulness for individualized clinical interpretation and study design. Drawing on contemporary guideline frameworks, systematic reviews, and international disease classification systems, this article highlights the limitations of static, anatomy-centered approaches and proposes an alternative conceptualization of cervical insufficiency as a dynamic, pregnancy-specific vulnerability. Within this framework, cervical behavior is understood as time-dependent and context-sensitive, shaped by the interplay of mechanical load, biological processes, and gestational timing rather than predetermined structural failure. This conceptualization is intended to inform interpretation across diverse clinical contexts, rather than to redefine diagnostic criteria or existing guideline recommendations. By shifting emphasis from fixed diagnostic labels to trajectories of cervical vulnerability, this Perspective situates cervical insufficiency within the broader continuum of spontaneous preterm birth and aligns its interpretation with the principles of personalized medicine. This conceptual reframing positions cervical insufficiency as a model condition for personalized maternal–fetal care, emphasizing time- and context-aware risk assessment and trajectory-informed clinical decision-making, while providing a coherent foundation for individualized surveillance and future research aimed at improving maternal–fetal outcomes.

## 1. Introduction: Beyond Terminology

Cervical insufficiency continues to occupy an ambiguous position in contemporary maternal–fetal medicine. Although the term has evolved to replace the pejorative notion of “cervical incompetence,” much of the underlying clinical reasoning remains anchored in a static, anatomy-centered model. This Perspective addresses a persistent conceptual gap between updated terminology and prevailing interpretive frameworks. This gap has tangible implications for clinical interpretation, surveillance strategies, and the design of preventive approaches, particularly in pregnancies that do not conform to traditional history-based risk profiles. Rather than revisiting nomenclature or proposing new diagnostic criteria, the focus is placed on how cervical insufficiency is understood and acted upon at the level of the individual pregnancy. By shifting attention from fixed labels to pregnancy-specific trajectories of cervical vulnerability, this article aims to provide a conceptual foundation for more personalized surveillance, risk stratification, and intervention strategies within the broader continuum of preterm birth.

Over the past two decades, the term cervical incompetence has gradually been replaced by cervical insufficiency in clinical guidelines and professional discourse. This linguistic shift reflects an important effort to avoid pejorative language and to acknowledge that cervical failure during pregnancy is not an absolute or binary defect. Instead, the term insufficiency implies gradation, context, and functional limitation rather than inherent inadequacy. However, this linguistic shift has not been accompanied by a corresponding transformation in the way cervical insufficiency is conceptualized or operationalized in clinical practice.

Yet, despite this terminological evolution, the conceptual framework underpinning cervical insufficiency has remained largely unchanged. Contemporary clinical reasoning continues to treat the condition as a pre-existing, fixed anatomic abnormality of the cervix, typically inferred retrospectively from obstetric history. As a result, dynamic cervical changes occurring within the index pregnancy may be under-recognized or overlooked until advanced stages. In practice, this assumption translates into rigid eligibility criteria for surveillance and intervention, most notably an emphasis on prior spontaneous preterm birth as a prerequisite for diagnosis and treatment.

This disconnect between updated terminology and persistent conceptual assumptions has practical consequences. It constrains the timing and intensity of surveillance, reinforces rigid thresholds for intervention, and limits opportunities for anticipatory risk stratification tailored to the evolving context of an individual pregnancy. It limits the explanatory power of existing models, fails to account for substantial clinical heterogeneity, and constrains the development of individualized preventive strategies. In an era increasingly defined by personalized medicine, such a static, anatomy-centered view appears increasingly insufficient.

Rather than asking whether a woman “has” or “does not have” cervical insufficiency, the more clinically relevant question is how cervical vulnerability evolves within a given pregnancy under specific biological, mechanical, and environmental conditions. By shifting attention from fixed labels to pregnancy-specific vulnerability, a more nuanced and personalized approach to maternal–fetal care may emerge. This shift in emphasis is not intended to redefine diagnostic criteria, but to recalibrate how cervical findings are interpreted over time within a specific pregnancy.

## 2. The Persistent Static–Anatomic Paradigm

Historically, cervical insufficiency has been conceptualized as a primarily structural vulnerability of the cervix that is assumed to predate pregnancy and becomes clinically apparent when exposed to gestational load. This anatomic paradigm, rooted in mid-20th-century surgical thinking, offered a clear and intuitively appealing explanation for painless cervical dilation and mid-trimester pregnancy loss, and provided a straightforward rationale for mechanical interventions aimed at supporting presumed structural deficiency. Despite subsequent advances in ultrasound surveillance and mechanistic insights into parturition, this structural framing has remained influential in how cervical insufficiency is described and acted upon in routine care.

Over time, this framework became embedded in contemporary clinical guidelines and routine practice, shaping how risk is defined, monitored, and acted upon across diverse clinical settings. Contemporary guideline frameworks continue to conceptualize cervical insufficiency primarily through a static, anatomy-centered lens, emphasizing prior spontaneous preterm birth and fixed diagnostic thresholds as the basis for surveillance and intervention strategies [1,2,3]. Within this approach, cervical competence is largely inferred retrospectively from obstetric history or prospectively from predefined measurement cutoffs, reinforcing a binary interpretation in which the cervix is considered either competent or insufficient. Such inference, while pragmatic, provides limited resolution for understanding cervical behavior as it unfolds within the index pregnancy. This binary framing leaves little conceptual space for intermediate or evolving states of vulnerability, particularly in patients who fall outside canonical history-based categories.

The prominence of this paradigm was further reinforced by landmark observational studies demonstrating an association between mid-trimester cervical length and the risk of spontaneous preterm birth [4]. Although these associations are robust and clinically valuable, they do not imply that cervical length alone captures the full spectrum of cervical behavior or vulnerability over time. These findings established cervical length measurement as a cornerstone of risk stratification and screening, supporting the use of a single static anatomic parameter as a surrogate for cervical competence. By design, such measurements offer a snapshot rather than a trajectory, privileging static structure over dynamic change. As a result, clinical decision-making has relied heavily on threshold-based assessments, with limited attention to temporal change or contextual modifiers. This emphasis can obscure clinically meaningful variation related to gestational timing, biological context, and pregnancy-specific mechanical load.

While this static–anatomic model offers practical advantages—simplicity, reproducibility, and ease of guideline implementation—it also imposes important conceptual constraints. These limitations have been increasingly recognized in contemporary reviews and guideline discussions addressing the multifactorial nature of preterm birth [5]. By privileging fixed structure over evolving behavior, it struggles to account for abrupt cervical change, pregnancy-to-pregnancy variability, or the influence of concurrent biological processes. Inflammatory signaling, extracellular matrix remodeling, and immune-mediated pathways are now understood to interact dynamically with cervical mechanics across gestation [6,7]. Consequently, the prevailing paradigm prioritizes classification over trajectory and structural assessment over dynamic interpretation, setting the stage for a growing mismatch between guideline-based reasoning and real-world clinical heterogeneity. This gap is most evident in clinical scenarios that do not conform to canonical history-based definitions yet nevertheless progress toward preterm birth [1].

## 3. Clinical Variability That Defies a Fixed-Defect Model

Although mid-trimester cervical length assessment has been widely adopted as a screening tool for preterm birth risk, systematic reviews have demonstrated that its predictive performance is modest and context-dependent, particularly in low-risk or asymptomatic populations [8]. Performance varies substantially according to baseline risk, gestational age, and the clinical context in which screening is applied. These limitations are readily apparent in everyday clinical practice, where patterns of cervical change frequently defy a fixed-defect model of cervical insufficiency. Such discordance between screening metrics and observed clinical trajectories challenges the assumption that cervical insufficiency reflects a stable, pre-existing anatomic defect.

One of the most striking examples is the presentation of advanced cervical dilation in nulliparous women with no prior history of pregnancy loss or preterm birth. Such presentations are difficult to reconcile with a model predicated on an antecedent structural defect of the cervix. Equally challenging is the marked variability observed across pregnancies within the same individual. This intra-individual variability underscores that cervical competence cannot be assumed to be fixed across pregnancies. Some women experience profound cervical change in one pregnancy yet carry subsequent pregnancies to term without intervention, while others demonstrate progressive cervical shortening only after a specific gestational window. The timing of cervical change, rather than its mere occurrence, appears to be a critical determinant of clinical trajectory. Such observations suggest a time-dependent susceptibility rather than a constant or pre-existing structural weakness.

In many cases, cervical change occurs in parallel with subclinical inflammation and membrane or decidual activation, blurring the boundary between cervical insufficiency and other pathways of spontaneous preterm birth. These processes may precede, accompany, or amplify cervical change, rather than simply result from it. Rather than representing an isolated mechanical failure, cervical remodeling may reflect downstream involvement in a broader inflammatory cascade that precedes both term and preterm labor [9]. This interaction highlights the limitation of attributing cervical change to a single dominant mechanism in isolation. In this context, cervical change appears less as a primary structural defect and more as a visible marker of broader biological processes unfolding during pregnancy. Viewed through this lens, cervical change functions as a dynamic indicator of pregnancy-specific vulnerability rather than a static anatomic diagnosis.

Importantly, cervical change is not always irreversible. Clinical experience indicates that timely intervention—whether mechanical, pharmacologic, or surveillance-based—can stabilize or slow progression in selected cases. These observations support an interpretive framework in which cervical behavior is modifiable over time, rather than predetermined by fixed anatomy. Such reversibility is difficult to reconcile with the notion of a purely fixed anatomic defect. They instead point toward a model in which vulnerability may fluctuate across gestation in response to timing, context, and intervention.

Taken together, these observations point toward a model in which cervical behavior is contingent, modulated, and pregnancy-specific. This model is intended to guide interpretation of cervical findings over time, rather than to redefine diagnostic categories. The cervix does not fail in isolation; rather, it responds dynamically to mechanical load, inflammatory signals, hormonal milieu, and gestational timing. These interacting dimensions help explain why similar anatomic findings may follow divergent clinical trajectories across pregnancies. Any framework that fails to incorporate this variability risks oversimplifying a complex and heterogeneous clinical phenomenon. Consistent with this view, recent systematic reviews synthesizing both prophylactic and emergency cerclage studies have highlighted substantial heterogeneity in patient selection, timing of intervention, and clinical outcomes, underscoring the limitations of a uniform, defect-based interpretation of cervical insufficiency [10]. Together, these findings reinforce the need to move beyond a singular structural explanation toward a trajectory-oriented understanding of cervical vulnerability.

## 4. From a Static Defect to a Dynamic Vulnerability

**Figure 1:**
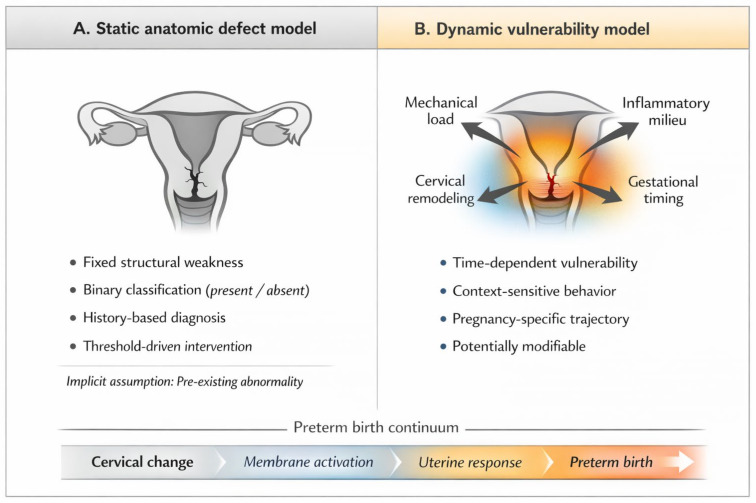
From fixed labels to pregnancy-specific trajectories of cervical vulnerability. (**A**) In the traditional static anatomic defect model, cervical insufficiency is conceptualized as a pre-existing, binary structural weakness of the cervix, inferred primarily from obstetric history or single-point measurements and managed using fixed diagnostic thresholds. Within this framework, cervical competence is classified categorically as either sufficient or insufficient, with limited consideration of temporal change or contextual modifiers. (**B**) In contrast, a dynamic vulnerability framework conceptualizes cervical behavior as time-dependent and context-sensitive, shaped by the interaction of mechanical load, inflammatory and biological processes, cervical remodeling, and gestational timing. Rather than representing a fixed diagnosis, cervical change is viewed as a pregnancy-specific trajectory embedded within the broader continuum of spontaneous preterm birth. This framework is intended as a conceptual schema to support individualized surveillance and contextual interpretation, rather than as a prescriptive diagnostic or management algorithm.

Reconceptualizing cervical insufficiency as a dynamic vulnerability rather than a static defect offers a more coherent explanation for the clinical heterogeneity observed across and within pregnancies. This reconceptualization is intended to refine interpretation rather than to redefine diagnostic categories or replace existing guideline frameworks. Vulnerability, in this context, does not deny the relevance of cervical anatomy; rather, it situates structural characteristics within a broader, time-dependent interplay of biological, mechanical, and contextual forces. These forces interact dynamically over time, accounting for the marked variability and reversibility described in clinical observation. Such a perspective shifts interpretation away from fixed labels toward pregnancy-specific trajectories of cervical behavior, emphasizing evolution over time rather than predefined categories. This vulnerability-based view aligns with broader models of spontaneous preterm birth that conceptualize it as a heterogeneous syndrome arising from multiple, often overlapping pathways rather than a single causative mechanism [11]. This shift from a label-based interpretation to a trajectory-oriented understanding of cervical behavior is illustrated in Figure 1. The figure is intended as a conceptual schema rather than a prescriptive algorithm.

A vulnerability-based model emphasizes that cervical competence is not a fixed property but an emergent state. This framing recognizes variability without presuming a uniform mechanism or a single dominant determinant across all patients. Mechanical stress from the growing uterus, biochemical remodeling of cervical connective tissue, inflammatory activation, and gestational timing collectively shape cervical behavior. Crucially, the influence of these factors may shift over gestation, altering vulnerability as pregnancy progresses. The relative contribution of each factor may vary between pregnancies and between individuals, producing diverse clinical trajectories that cannot be captured by static classification alone. As a result, static categories may obscure clinically meaningful patterns that emerge only when cervical change is interpreted longitudinally.

Crucially, vulnerability is conditional rather than constant. A cervix that remains stable under one set of circumstances may become susceptible under another, depending on timing, context, and concurrent biological processes. This perspective shifts clinical attention from isolated measurements to evolving patterns—how rapidly cervical change occurs, under what conditions dilation emerges, and how the cervix responds to intervention once vulnerability becomes apparent.

Such a framework integrates cervical insufficiency naturally into the broader continuum of preterm birth. Rather than treating cervical insufficiency as a discrete entity, it is better understood as one manifestation of pregnancy-specific vulnerability within a complex, multistep process. Within this continuum, cervical change may serve as an early or visible marker of underlying pathophysiological activity rather than as an isolated primary cause.

By reframing cervical insufficiency as dynamic vulnerability, the clinical focus moves from labeling to anticipation. The central questions become when vulnerability emerges, how it progresses over time, and how evolving patterns of cervical change may inform individualized clinical interpretation. This conceptual shift provides the foundation for personalized surveillance strategies and more flexible decision-making thresholds, setting the stage for the implications discussed in the following section.

## 5. Implications for Personalized Maternal–Fetal Care

Viewing cervical insufficiency through the lens of dynamic vulnerability has direct implications for how personalized maternal–fetal care is conceptualized and operationalized in clinical practice. Risk stratification can move beyond binary, history-based criteria toward a more nuanced state- and time-aware assessment that incorporates gestational timing, cervical dynamics, inflammatory context, and pregnancy-specific patterns of change. Such an approach recognizes that vulnerability is not uniformly present throughout gestation, and that it cannot be reliably inferred from a single prior obstetric event.

From this perspective, surveillance becomes the central mechanism through which a vulnerability-based framework is translated into personalized maternal–fetal care, allowing risk to be continuously reassessed rather than assigned once. Surveillance strategies can therefore be individualized according to anticipated windows of susceptibility, rather than dictated by uniform screening schedules or fixed diagnostic thresholds that implicitly assume stable risk across gestation. By aligning monitoring with the temporal evolution of cervical change, this approach enables earlier recognition of emerging risk in pregnancies that might otherwise be labeled as low risk. Crucially, such labeling is reframed as provisional and revisable, reflecting the principle that the absence of a prior spontaneous preterm birth does not reliably indicate the absence of vulnerability in the current pregnancy. This trajectory-oriented approach is supported by individual patient-level meta-analytic evidence demonstrating that cerclage reduces preterm birth in singleton pregnancies with a short cervix identified before 24 weeks, even in the absence of a prior spontaneous preterm birth [12].

Intervention strategies likewise benefit from a trajectory-oriented framework. Mechanical support, pharmacologic modulation, and expectant management can be considered in response to observed patterns of progression rather than predefined diagnostic categories alone. In this context, intervention is guided by when and how vulnerability evolves, not simply by what label is assigned. The clinical objective shifts from correcting a presumed structural defect to stabilizing a vulnerable system at a critical moment in gestation.

Importantly, a vulnerability-based framework promotes integration rather than fragmentation of care. Cervical findings are interpreted alongside uterine activity, membrane status, inflammatory signals, and broader obstetric context, supporting a more holistic approach to preterm birth prevention. In this sense, cervical insufficiency serves as a test case for personalized medicine in maternal–fetal care, illustrating how attention to timing, context, and trajectory can enhance both clinical reasoning and responsiveness.

## 6. Conclusions: From Labels to Trajectories

The transition from cervical incompetence to cervical insufficiency marked an important linguistic correction, but it did not, by itself, resolve deeper conceptual limitations. Continued reliance on a static, anatomy-centered model constrains our ability to explain the wide clinical variability observed in practice and limits opportunities for individualized care.

By shifting focus from fixed labels to pregnancy-specific trajectories of vulnerability, cervical insufficiency can be more coherently integrated into the broader continuum of preterm birth. This perspective emphasizes that cervical behavior is time-dependent, context-sensitive, and potentially modifiable, rather than predetermined by a single structural attribute.

Notably, even the transition from ICD-10 to ICD-11 has not been accompanied by a comprehensive reconceptualization of cervical insufficiency within international disease classification systems. This reflects the primary function of ICD as an epidemiologic and administrative tool rather than a vehicle for pathophysiologic refinement, underscoring the persistence of legacy, defect-oriented constructs despite evolving clinical understanding [13].

Ultimately, the critical question is no longer whether cervical insufficiency exists as a discrete diagnostic entity, but how cervical vulnerability unfolds within each pregnancy—and how clinicians can recognize, interpret, and respond to that process in a timely and individualized manner. Such a conceptual shift aligns naturally with the principles of personalized medicine and provides a framework for future research and clinical strategies aimed at improving outcomes in maternal–fetal care.

## Data Availability

No new data were created or analyzed in this study. Data sharing is not applicable to this article.

## Abbreviation

ICD	International Classification of Diseases
